# A unique tripartite collision tumor of the esophagus: A case report

**DOI:** 10.1097/MD.0000000000008784

**Published:** 2017-12-08

**Authors:** Dimitrios Schizas, Adamantios Michalinos, Paraskevi Alexandrou, Demetrios Moris, Evangelia Baliou, Diamantis Tsilimigras, Theodore Throupis, Theodore Liakakos

**Affiliations:** a 1st Department of Surgery; b Department of Pathology, National and Kapodistrian University of Athens, Athens, Attiki, Greece; c Division of Surgical Oncology, The Ohio State University Wexner Medical Center and James Cancer Hospital and Solove Research Institute, Columbus, OH; d National and Kapodistrian University of Athens; e Department of Anatomy, National and Kapodistrian Athens, Athens, Attiki, Greece.

**Keywords:** collision tumor, esophageal cancer, mixed adeno-endocrine neoplasm, neuroendocrine neoplasm, tripartite neoplasm

## Abstract

**Rationale:**

We report a unique case of a tripartite esophageal collision tumor consisting of three separate histologic types.

**Patients concerns:**

Therapeutic dilemmas on the proper treatment of those rare neoplasms remain unanswered considering both proper surgical therapy and adjuvant therapy.

**Diagnose:**

In this paper, we report a unique case of a patient with a tripartite esophageal collision tumor consisting of a small cell carcinoma, an adenocarcinoma of medium differentiation and a signet ring cell carcinoma. Diagnosis is difficult as clinical presentation of the patient was undistinguishable from other, commoner tumor types.

**Interventions:**

The patient's diagnostic and therapeutic course along with available data on the collisions tumor's biological behavior and treatment are briefly discussed.

**Outcomes:**

Esophagectomy is the best treatment options for these patients. Unique nature of this tumor demands aggresive oncologic treatment.

**Lessons:**

Collision tumors are rare neoplasms consisting of distinct cell populations developing in juxtaposition to one another without any areas of intermingling. Various cell types can be found. However, collision neoplasms of the esophagus combining adenomatous and neuroendocrine components are exceedingly rare, with only 5 cases described to date in the literature. Given their rarity, limited information is available on their tumorigenesis, biological behavior and clinical course. In general, these tumors are aggressive neoplasms and significantly affect patient treatment and prognosis.

## 1 Introduction

Collision tumors are rare neoplasms consisting of 2 cell populations developing in juxtaposition to one another without any or only minimal areas of intermingling.^[1]^ These tumors are part of the family of neoplasms that consist of 2 cell populations. Additional tumor categories include composite tumors, carcinosarcomas, and amphicrine tumors.^[2]^ Collision tumors of the esophagus with an adenocarcinoma and neuroendocrine component are exceedingly rare, with only 5 cases described to date. The tumorigenesis of those neoplasms has largely been uninvestigated; however, genetic studies indicate that these tumors originate from a common malignant progenitor cell that differentiates into 2 distinct cell types that maintain their malignant characteristics.^[3,4]^

Given their rarity, their biologic behavior remains largely unknown, with the only available data originating from case reports and small case series. The existence of these tumors has significant implications on patient treatment as they are aggressive neoplasms with high malignant potential.^[3]^

In this report, we describe a case of a tripartite esophageal collision tumor with a small cell carcinoma (SCC), medium differentiated adenocarcinoma, and signet ring cell carcinoma. Tripartite neoplasms of the esophagus are exceedingly rare, with only few cases described to date. Our case is unique in terms of the collision of 3 instead of 2 components and tripartite differentiation into a combination not previously described to date.

## 2 Case report

A 76-year-old Caucasian male patient presented at our department with progressive dysphagia.

The patient's symptoms appeared 4 months before his admission when he first noticed dysphagia to solid food. During this period, he lost approximately 15 kg. He had a known history of gastroesophageal reflux disease under medical treatment. He had not undergone an endoscopy for the past 5 years. The remainder of his medical history was unremarkable.

At admission, the patient presented mild cachexia and could only tolerate a liquid diet. The physical examination and laboratory examinations were unremarkable. His carcinoembryonic antigen value was 0.04 g/L and Cancer Antigen 19-9 was 0.451 g/L. The patient underwent an upper gastrointestinal endoscopy that revealed Barret esophagus, extending from the cardia of the stomach to the upper esophagus. Two pedunculated lesions, including 1 at the mid-esophagus and the other at the cardia of the stomach, were also noted. Biopsies from the upper nodule were inconclusive. Biopsies from the lower nodule revealed an adenocarcinoma of low differentiation. The patient was staged with a computerized tomography scan that revealed diffuse thickening of the esophageal wall. Regional or distant lymphadenopathy and distant metastases were not noted.

**Figure 1:**
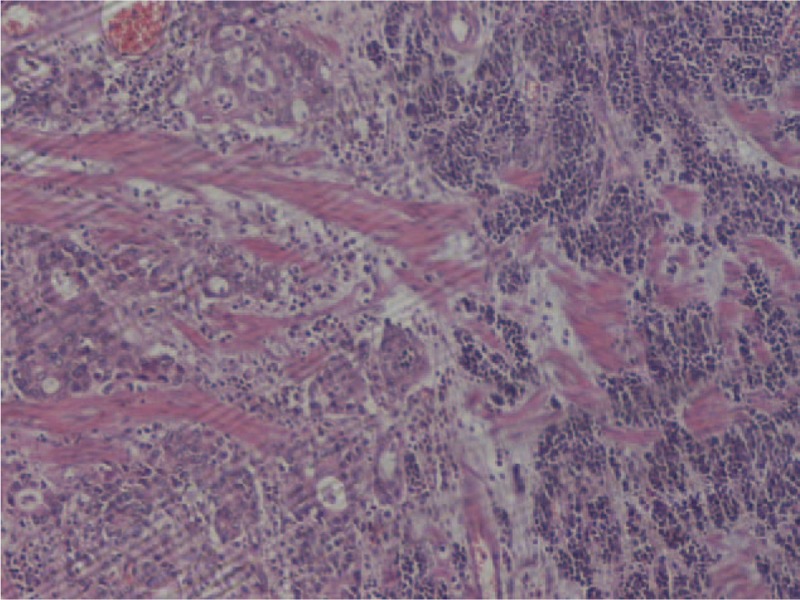
Collision tumor of a mixed enteric type adenocarcinoma and small cell carcinoma. H&E ×100.

**Figure 2:**
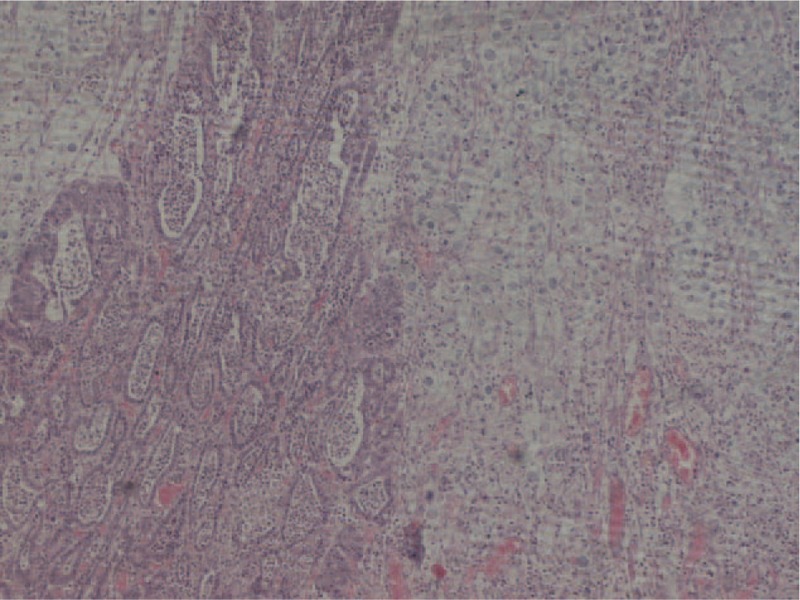
Collision tumor of a moderately differentiated enteric type adenocarcinoma and signet ring adenocarcinoma. H&E ×100.

**Figure 3:**
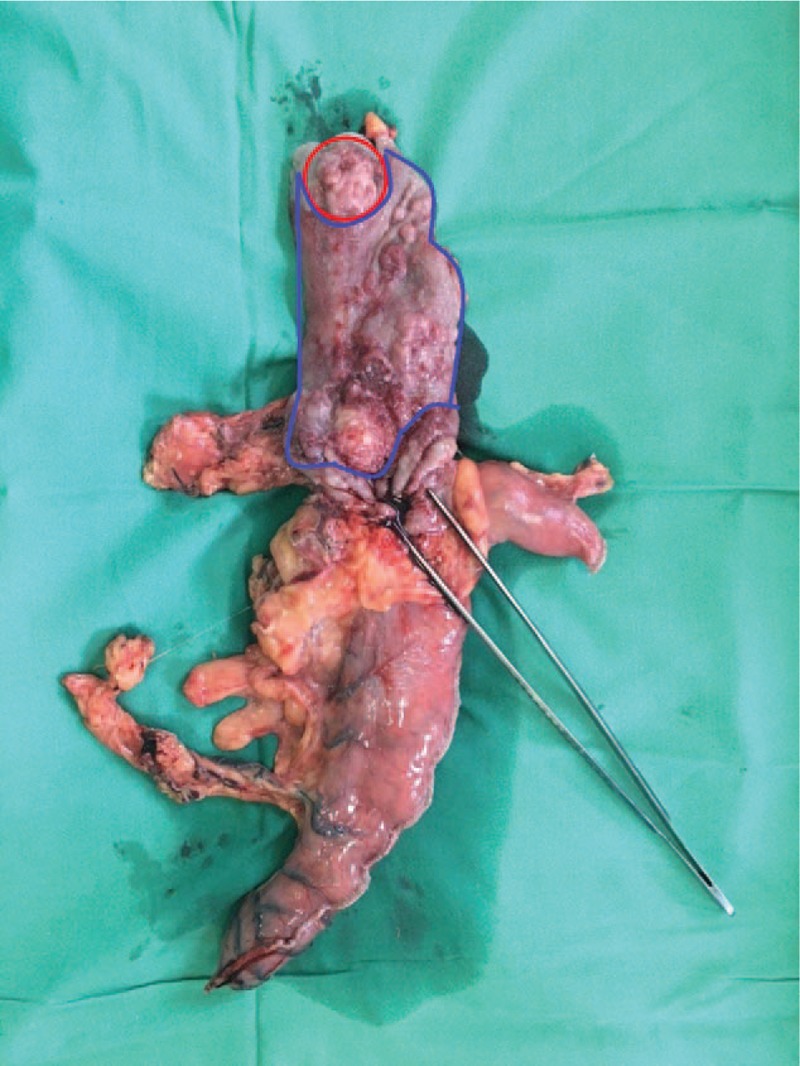
Three histologic types of the tumor colliding in pairs are noted in the specimen. Red: moderately differentiated enteric type adenocarcinoma and neuroendocrine tumor. Blue: moderately differentiated type adenocarcinoma and signet ring adenocarcinoma.

With the provisional diagnosis of adenocarcinoma of the gastroesophageal junction and extended Barret esophagus, the patient underwent a transthoracic total esophagectomy with standard lymphadenectomy. His postoperative course was uneventful, and he left the hospital on the 15th postoperative day. Histological examination of the specimen revealed a large tumor extending from the upper surgical border to the stomach with a significant submucosal extension. In total, 35 lymph nodes were excised. The neoplasm extended to the muscular layer of the esophagus without infiltrating the periesophageal fat. Histologically, the tumor consisted of a moderately differentiated adenocarcinoma abutting a small cell carcinoma (upper nodule) (Fig. 1) and a poorly cohesive adenocarcinoma (lower nodule) in a colliding manner (Fig. 2) The 3 histologic types of the tumor formed a single tumor that had 2 nodules and a significant submucosal expansion (Fig. 3). One lymph node was infiltrated by the moderately differentiated adenocarcinoma. The tumor was staged as PT3N1MO.

Considering the aggressive neuroendocrine component of the tumor, the patient received an adjuvant therapy of cisplatin and etoposide. After 6 months of follow-up, he presented with multiple liver metastases. Since then, he has received definite chemotherapy.

## 3 Discussion

Collision tumors are rare neoplasms consisting of 2 distinct cell populations developing in juxtaposition to one another with or without minimal areas of intermingling. A collision tumor is a subtype of neoplasms consisting of 2 distinct cell populations, and other types include composite tumors (no clear-cut interface or a transition zone between histological patterns) and carcinosarcomas (extensive intermingling between cell populations). Amphicrine neoplasms (1 cell population exhibiting characteristics of both epithelial and sarcomatous) and cancer-to-cancer metastasis are also included in these rare neoplasms.^[1,2]^ Spagnolo and Heenan^[5]^ proposed diagnostic criteria for the definition of a collision tumor: (1) 2 distinct topographically separate sites of origin for the 2 components must be present; (2) there must be at least some separation of the 2 components, such that despite intimate mixing at points of juxtaposition, a dual origin can be recognized; and (3) at the areas of the collision, various transitional patterns may be observed in addition to an intimate mixing of the 2 components, such as a mucoepidermoid appearance in the case of a collision between squamous cell carcinoma (SqC) and adenocarcinoma. Various collision carcinomas have been described in the esophagus, including SqC and adenocarcinoma,^[6]^ SqC and gastrointestinal stromal tumors,^[7]^ and SqC and neuroendocrine carcinoma.^[8]^ Collision tumors consisting of adenocarcinoma and neuroendocrine carcinoma are exceedingly rare, with no more than 5 cases described in the literature (Table 1). Notably, all patients described to date are male, and the most common neuroendocrine component is SCC. Purdy and Gaffney^[9]^ also reported that 2 lymph nodes that were excised in their case were exclusively invaded by the adenocarcinoma component. Our case is unique compared with those described in the literature, given that, to the best of our knowledge, no tripartite collision tumor of any combination has been described.

**Table 1:**
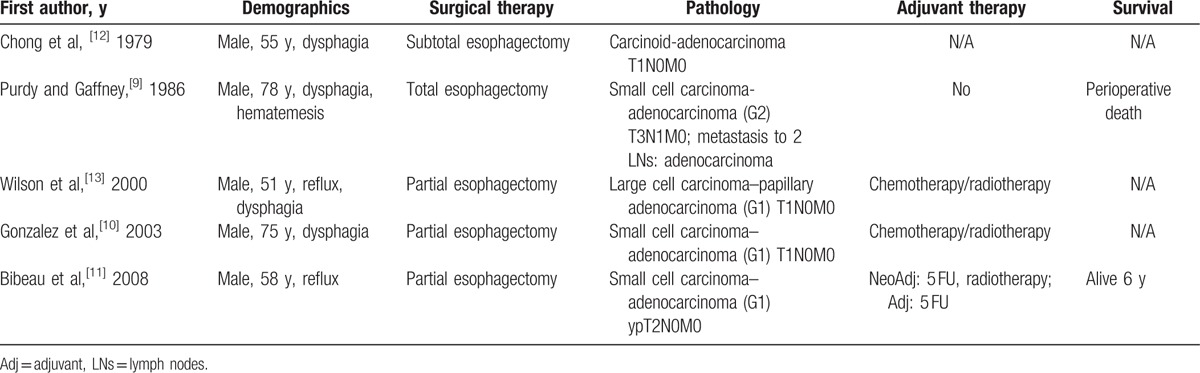
Collision neuroendocrine–adenocarcinoma neoplasms.

Various theories have been proposed for tumorigenesis of these rare neoplasms, including the accidental meeting of 2 coexisting neoplasms—common carcinogen theory and stimulated tumor-to-tumor carcinogenesis.^[2]^ A shared component of these theories involves the acceptance of the biclonality of those neoplasms. However, genetic data indicate that collision tumors originate from a single cell that later differentiates into 2 distinct cell populations. Milne et al^[3]^ performed p53 and loss of heterozygosity (LOH) analyses on 2 collision and 3 composite tumors of SqC–adenocarcinoma of the gastroesophageal junction and found shared p53 mutations and common LOH patterns. Fukui et al^[4]^ performed the same study on a neuroendocrine carcinoma–adenocarcinoma collision tumor of the stomach and obtained similar results. These authors proposed that a common progenitor cell underwent a malignant transformation and later differentiated into different malignant cell types. On the contrary, Iwaya et al^[14]^ performed LOH analysis on a carcinosarcoma of the esophagus and found different LOH patterns between various components of the tumor. In the singe case of a neuroendocrine carcinoma–adenocarcinoma of the esophagus with available genetic data,^[10]^ different LOH patterns were observed between the neuroendocrine and adenocarcinoma components. Adachi et al^[15]^ proposed that pluripotent stem cells in the esophageal endoderm can undergo a malignant transformation independently and synchronously. This discrepancy in the literature indicates that not all collision tumors follow the same tumorigenesis processes. It is possible that the genetic alterations investigated occurred after differentiation of a malignant common progenitor cell into different cell types, which is thus not present in both cell populations.

Collision tumors of neuroendocrine-adenocarcinomas can also be considered to be extreme forms of mixed adeno-neuroendocrine carcinomas (MANECs) given that they fulfil their diagnostic criteria. In some MANECs, the neuroendocrine and exocrine components develop in separate areas of the same lesion (composite or collision neoplasms). By contrast, in other MANECs, the components are intimately and diffusely admixed (combined neoplasms).^[16]^ Genetic studies performed in MANECs indicate a common cell origin, given that they share common genetic mutations. Specifically, common LOH patterns between 2 components indicate a multistep progression from a common precursor lesion, with different mutations occurring later during tumorigenesis.^[17]^ In fact, neuroendocrine neoplasms share common oncogenetic pathways with adenocarcinomas and may correspond to high-grade transformation.^[18]^

Tripartite neoplasms have rarely been described in the esophagus.^[19–22]^ Nishimaki et al^[19]^ described a tripartite neoplasm of SqC, adenocarcinoma, and SCC. Notably, the excised lymph nodes were infiltrated by an admixture of adenocarcinoma and SCC. Pai et al^[20]^ described a tripartite neoplasm consisting of SqC, adenocarcinoma, and leiomyoma, with the lymph nodes only invaded by SqC. Kanamoto et al^[21]^ described a case with multidirectional differentiation of neuroendocrine SqC ciliated glandular and sarcomatous components, whereas Tanabe et al^[22]^ described 3 cases of composite neoplasms with SqC, adenocarcinoma, and SCC. In the aforementioned cases, the squamous cell component formed the superficial parts of the lesion, whereas adenocarcinoma and small cell components formed the deeper parts. For those rare cases, it has been proposed that these lesions derive from a common pluripotential or totipotential progenitor cell that differentiates later during carcinogenesis under unknown genetic or environmental factors.^[19–21]^ Our case is also unique in this context, given that, to the best of our knowledge, no tripartite esophageal neoplasm involving SCC, signet ring cell carcinoma, and adenocarcinoma has been described to date.

Clinically, these neoplasms are indistinguishable and preoperative diagnosis is rare, given that they present no special clinical or radiological features. A preoperative diagnosis can be established only incidentally as biopsy typically involves only 1 of the 2 components of the tumor.^[10,18]^ However, their existence significantly alters treatment options as it affects the adjuvant treatment option. Given the rarity of collision tumors, every case should be treated in an individualized manner. No established guidelines are available. However, in general, treatment should target the more aggressive component.^[16,19]^ SCC of the esophagus is a rare yet aggressive neoplasm that comprises 0.5% to 4% of esophageal cancer.^[23]^ According to recent guidelines,^[24]^ aggressive therapy with cisplatin and carboplatin with etoposide is recommended. According to Bibeau et al,^[11]^ combined therapy targeting both tumor components can also be considered.
